# 

**Published:** 1878-12

**Authors:** 


The New York Medical Record, The Boston Medical and Surgical Journal, The Medical and Surgical Reporter, The Cincinnati Lancet and Clinic, The Louisville Medical News, The Hospital Gazette, The Journal of Materia Medica, The Illinois Medical Recorder, The Nashville Journal of Medicine and Surgery, The Medical News and Library, The Monthly Abstract of Medical Sciences, The New Remedies, The New York Eclectic Medical and Surgical Journal, The Chicago Medical Journal and Examiner, The St. Louis Medical and Surgical Journal, Proceedings of the Medical Society of the County of Kings, The Eclectic Medical Journal, The Obstetric Gazette, The Pharmacist, The American Practitioner, New Preparations, Chicago Journal of Nervous and Mental Diseases, Archives of Dermatology, London Lancet, (Wm. C. Herald, Publisher), Dental Cosmos, Journal of Industry, Review and Index, The Laboratory, The New England Medical Gazette, The Cincinnati Medical News, The Cincinnati Medical Advance, Monthly Journal Illinois Medical Association, Medical Examiner and Chicago Medical Journal, Detroit Lancet, American Observer, Quarterly Journal of Inebriety (Hartford), Medical Review (Indianapolis), Kansas City Medical Journal, The American Practitioner, American Medical Weekly, Arkansas Medical Record, Braithwaite's Retrospect, Hall's Journal of Health, Druggists' Circular, Herald of Health, American Journal of Obstetrics, American Agriculturist, Archives of Opthalmology and Otology, American Newspaper Reporter, Archives of Electrology and Neurology, The Lancet, Physician and Pharmacist, New York Observer, New York Medical Brief, Journal Materia Medica, Boston Journal of Chemistry, The American Journal of Dental Science, The Ohio Medical and Surgical Journal, The Maryland Medical Journal, The American Journal of Pharmacy, The Virginia Medical Monthly, The Obstetrical Journal, The St. Louis Clinical Record, The Philadelphia Medical Times (biweekly), The Toledo Medical and Surgical Journal, The North Carolina Medical Journal, The American Medical Journal, The New York Medical Journal, The Michigan Medical News, The Southern Medical Record, The Physio-Medical Journal, Atlanta Medical and Surgical Journal, Ohio Medical Recorder, Richmond and Louisville Medical Journal, American Medical Bi-Weekly, United States Investigator, Sanitarian, New Orleans Medical and Surgical Journal The Iowa Catlin, American Journal Medical Science, Half-Yearly Compendium Medical Sciences, The Medical Times, Transactions of College of Physicians, Obstetrical Journal, of Great Britain and Ireland, Hahnemanian Monthly, Druggist and Chemist, Lersine and Monthly Bulletin, Northern Tier Reporter, Virginia Clinical Record, Missouri Dental

Journal, Pacific Medical Journal, The Western Lancet, The Medical Recorder, Journal of Insanity.

*Foreign Journals.*—L'Union Medicale du Canada, Canada Journal of Medical Science, Canada Medical and Surgical Journal, Canada Medical Record, Canada Health Journal. ENGLAND.—Chemist and Druggist, The Doctor, The Medical Press and Circular, The Medical Record.

Diseases of the Bladder and Urethra in Woman. By Alexander J. C. Skene, M. D. Wm. Wood & Co , New York. H. H. Otis, Buffalo.

On Rest and Pain: A course of Lectures on the Influence of Mechanical and Physical rest in the treatment of Accidents and Surgical Diseases, and the Diagnostic value of Pain. By John Hilton, F. R. S., F. R. C. S. Edited by W. H. A. Jacobson, F. R. C. Wm. Wood & Co. New York.

Notes on the Treatment of Skin Diseases. By Robert Liveing, A. M., M. D. Wm. Wood & Co. New York. H. H. Otis, Buffalo.

Practical Surgery; including Surgical Dressings, Bandaging, Ligations and Amputations. By J. Ewing Mears, M. D. Lindsay & Blakiston, Philadelphia. T. H. Butler, Buffalo.

Clinical Diagnosis: A Hand-Book for Students and Practitioners of Medicine. By James Finlayson, M. D. Henry C. Lea, Philadelphia. T. H. Butler, Buffalo.

A History of the Diagnosis, Pathology and Treatment of Yellow Fever. By J. B. Marvin, M. D.

Case of Sarcoma of the Kidney of a Negro Child. By W. H. Geddings, M. D., Aiken, S. C.

Uterine Pathology and Treatment. By R. E. Beach, M. D., Vandalia, Ill.

A Therapeutical Inquiry into Rational Medicine. By S. W. Wetmore, M. D., Buffalo, N. Y.

A Series of American Clinical Lectures. Edited by E. C. Seguin, M. D., on the treatment of the various forms of Acne and of Rosacea. By R. W. Taylor, M. D.

The Local Treatment of Eczema. By Henry G. Piffard, M. D.

The Constituents of Climate, with Special Reference to the Climate of Florida. By Frederick D. Lente, A. M., M. D.

Report of the Buffalo Eye and Ear Infirmary.

Case of Poisoning by oil of Chenopodium. By Thomas R. Brown, M. D.

Biographical Sketch of William M. Knight, M. D., Ex-President of the Society. Read before the Philadelphia Co. Medical Society, by Henry H. Smith, M. D.

Address of Erastus C. Benedict, L.L. D., Chancellor of the University of the State of New York, delivered at Albany, N. Y., July 9, 1878.

Clinical Lectures on Surgery delivered at Starling Medical College. By J. H. Pooley, M. D.

Artificial Feeding of Infants, and description of Instruments and Apparatus of the Author, with directions for their use. By A. Clendinen, M. D., Fort Lee, N. J.

The Eight Annual Report of the Board of Directors of the Free Medical and Surgical Dispensing Association of the City of Buffalo.

THE BUFFALO

### MEDICAL AND SURGICAL JOURNAL.

### MEDICAL AND SURGICAL JOURNAL.

PUBLISHED MONTHLY,—Containing Original Articles, Reports of Medical Societies and Hospitals, Editorial, Reviews, Correspondence, Army News, etc., etc; including the usual variety of Medical Periodical Publications. Specimen copies sent on application to BUFFALO MEDICAL AND SURGICAL JOURNAL, J. F. MINER, M. D., 978 Main Street, BUFFALO, N. Y.

TERMS OF SUBSCRIPTION, $3 00 PER YEAR, IN ADVANCE,—All communications for publication should be sent before the fifteenth (15) of each month. or they will of necessity lie over until the next number. No change can be made in advertisements after the twenty-fifth.

Correspondence and original articles are solicited. Publication is no evidence that the views of the author are endorsed by the Journal.

